# Ontogenetic convergence and evolution of foot morphology in European cave salamanders (Family: Plethodontidae)

**DOI:** 10.1186/1471-2148-10-216

**Published:** 2010-07-16

**Authors:** Dean C Adams, Annamaria Nistri

**Affiliations:** 1 Department of Ecology, Evolution, and Organismal Biology, and Department of Statistics, Iowa State University, Ames IA, 50011, USA; 2 Museo di Storia Naturale, Sezione di Zoologia "La Specola", Universitá di Firenze, Firenze, 50125, Italia

## Abstract

**Background:**

A major goal in evolutionary biology is to understand the evolution of phenotypic diversity. Both natural and sexual selection play a large role in generating phenotypic adaptations, with biomechanical requirements and developmental mechanisms mediating patterns of phenotypic evolution. For many traits, the relative importance of selective and developmental components remains understudied.

**Results:**

We investigated ontogenetic trajectories of foot morphology in the eight species of European plethodontid cave salamander to test the hypothesis that adult foot morphology was adapted for climbing. Using geometric morphometrics and other approaches, we found that developmental patterns in five species displayed little morphological change during growth (isometry), where the extensive interdigital webbing in adults was best explained as the retention of the juvenile morphological state. By contrast, three species exhibited significant allometry, with an increase in interdigital webbing during growth. Phylogenetic analyses revealed that multiple evolutionary transitions between isometry and allometry of foot webbing have occurred in this lineage. Allometric parameters of foot growth were most similar to those of a tropical species previously shown to be adapted for climbing. Finally, interspecific variation in adult foot morphology was significantly reduced as compared to variation among juveniles, indicating that ontogenetic convergence had resulted in a common adult foot morphology across species.

**Conclusions:**

The results presented here provide evidence of a complex history of phenotypic evolution in this clade. The common adult phenotype exhibited among species reveals that selection plays an important part in generating patterns of foot diversity in the group. However, developmental trajectories arriving at this common morphology are distinct; with some species displaying developmental stasis (isometry), while others show an increase in foot webbing during growth. Thus, multiple developmental solutions exist to the same evolutionary challenge. Our findings underscore the importance of examining morphological adaptations from multiple perspectives, and emphasize that both selective hypotheses and developmental processes must be considered for a more comprehensive understanding of phenotypic evolution.

## Background

How can we explain the extent of phenotypic diversity observed in nature? Since Darwin, a myriad of studies have proposed adaptive explanations for phenotypic variation, and have focused on the role of natural and sexual selection in shaping patterns of diversification. For instance, divergent selection often generates discontinuities among populations and species, enhancing adaptive differences through time [1-4]. Additionally, common selective pressures found in distinct locations can generate similar patterns of morphological evolution among unrelated groups, resulting in evolutionary convergence [5-8] or parallelism [9-12]. These examples, and many others, provide strong evidence of adaptation, and speak to the power of selection in directing morphological change [13].

While phenotypic variation is often assumed to be adaptive and molded by natural selection, several additional mechanisms play important roles in influencing and constraining morphological change [14,15]. For example, functional and biomechanical requirements can restrict the evolutionary response to selection, particularly when competing functional demands on the same trait cannot be simultaneously optimized [16,17]. Likewise, patterns of genetic covariance [18-20] and underlying developmental pathways [21,22] can alter both the direction and extent of morphological change, and influence the degree to which selection can operate. Such structural mechanisms commonly interact with selection to shape the course of evolution, and while phenotypic traits are often portrayed as being the result of either selection or constraints, a full appreciation of the evolutionary process requires understanding the contributions of both components [23].

In some instances, the mechanisms responsible for the evolution of phenotypic traits commonly considered to be adaptive are more intricate than was previously believed. For example, many tropical plethodontid salamanders are arboreal [24,25], and as adults, have extensive webbing on their hands and feet. Because this interdigital webbing has evolved repeatedly in the group, it was long hypothesized that this derived trait was an adaptation to their arboreal habit [24,26]. Indeed, an ontogenetic analysis revealed that webbed and non-webbed *Bolitoglossa *species diverge in their developmental trajectories, with webbed species maintaining a high degree of webbing throughout growth (paedomorphosis), while non-webbed species attained less webbing as adults [27]. Interestingly however, an analysis using a biomechanical model for foot growth [26] revealed that webbed and non-webbed species shared a common pattern of increased foot area relative to body size. Thus, it was concluded that foot webbing was not an adaptation for climbing in these species [27], as the high-degree of webbing did not result in increased foot area in the webbed species (the critical parameter for successful climbing: see [26]). Only a single webbed species (the cave-dwelling *Chiropterotriton magnipes*) had a growth trajectory where foot area increased relative to body size in a manner consistent with adaptation. This example demonstrates the importance of considering both selective and structural mechanisms when assessing patterns of morphological change.

The European plethodontid salamanders provide an interesting opportunity to examine the relative influence of selection and development on patterns of morphological evolution. These species are part of a larger clade (*Hydromantes*) inhabiting North America and Europe [28,29]. The eight European species diverged from their North American counterparts during the Eocene [29-31], and based on their disjunct distribution and their monophyly, some refer to the European lineage as a separate genus, *Speleomantes *[31,32] [some authors have placed species of *Hydromantes *in one of several subgenera [28,33]; here we refer to all European species as *Hydromantes (Speleomantes*)]. European *Hydromantes *are direct-developing amphibians (i.e. they display no larval stage), and frequently inhabit caves and crevices, where they can be found climbing the walls and ceilings [30,34,35]. In more terrestrial environments, they often cling to rock faces, walls, and trees [31,36-38]. As adults, these species display considerable webbing on their hands and feet, a morphological trait believed to be an adaptation for climbing [31,34]. However, the degree of foot webbing has not been examined throughout the course of development in these species. Thus, it remains unknown whether patterns of foot webbing in adults are the result of adaptation, as the hypothesis has not been formally tested. The purpose of this paper is to explore the ontogenetic trajectories of foot morphology in European *Hydromantes *to determine what forces may have shaped patterns of morphological evolution. Specifically, we test the hypothesis that adult foot morphology represents an adaptive condition, as previously proposed [31,34]. We also examine ontogenetic patterns in light of the phylogenetic history of the group, to examine the extent to which developmental processes influence patterns of adult foot morphology [28].

## Results

**Figure 1:**
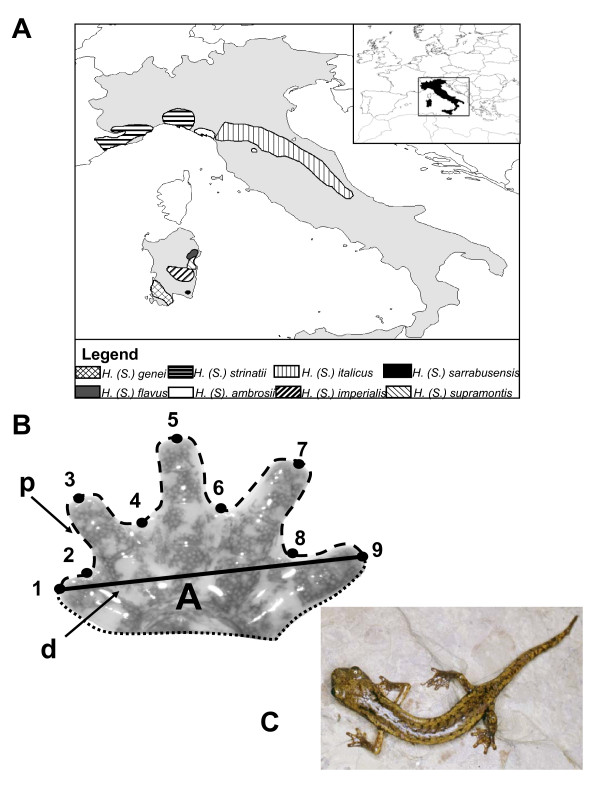
**A) Geographic distributions of all species of European *Hydromantes*; B) Measurements used to characterize foot morphology; C) photo of *Hydromantes (S.) strinatii *(courtesy of S. Vanni)**. Foot morphology in European *Hydromantes *was quantified in several ways, following [27]: 1) foot shape, as defined by the positions of nine anatomical landmarks (numbered), 2) the degree of foot webbing, found as the ratio between the perimeter of the foot (p) divided by foot width (d), 3) foot area (A) enclosed by the outline of the entire foot. The species examined in this study are: *H. (S.) ambrosii, H. (S.) flavus, H. (S.) genei, H. (S.) imperialis, H. (S.) italicus, H. (S.) sarrabusensis, H. (S.) strinatii*, and *H. (S.) supramontis*.

**Figure 2:**
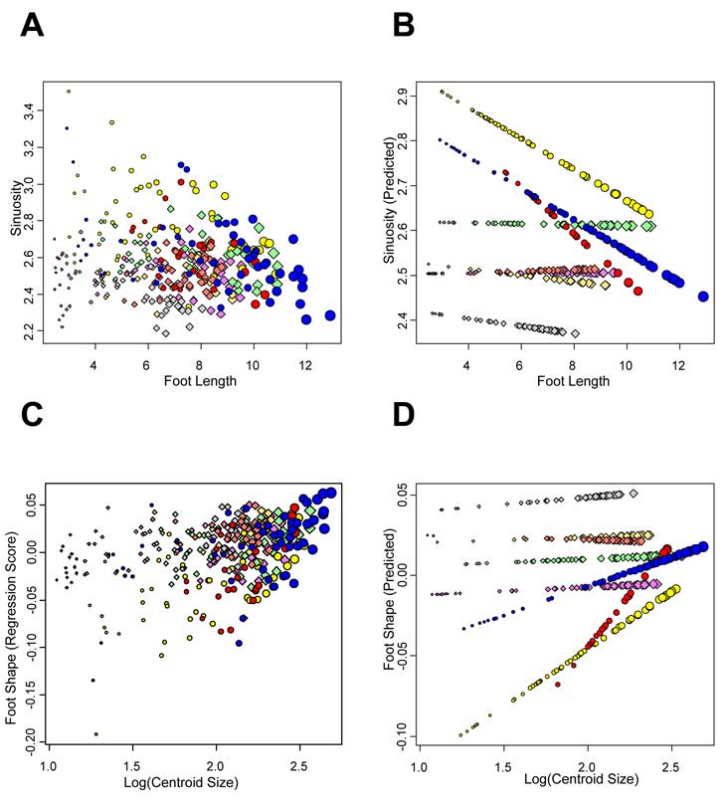
**Measures of foot morphology as a function of size for all species of European *Hydromantes***. A) Foot sinuosity versus foot length, B) Predicted values of foot sinuosity ($\hat{Y}$) from species-specific regressions versus foot length, C) Regression scores of foot shape [46] versus log(Centroid Size), D) Predicted values of foot shape ($\hat{Y}$) from species-specific regressions versus log(Centroid Size). In all panels, symbol size is proportional to specimen size. Species displaying allometric relationships are shown as circles, while species displaying isometric relationships are shown as diamonds. Species are shown in the following colors: *H. (S.) ambrosii *= violet; *H. (S.) flavus *= yellow; *H. (S.) genei *= beige; *H. (S.) imperialis *= light green; *H. (S.) italicus *= light gray; *H. (S.) sarrabusensis *= red; *H. (S.) strinatii *= salmon; *H. (S.) supramontis *= blue.

**Figure 3:**
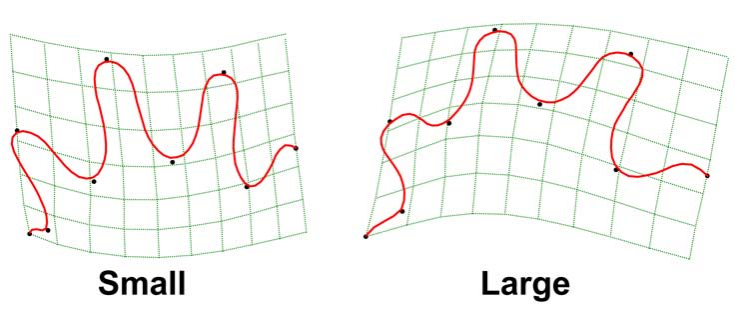
**Thin-plate spline deformation grids depicting foot shape for A) small and B) large individuals**. Grids are accentuated by a factor of two to facilitate visual interpretation.

We quantified foot morphology from juvenile and adult specimens of all eight species of European *Hydromantes *native to continental Italy and Sardinia (Figure 1A; species listed in Figure legend). Foot morphology was characterized using a variety of measures (Figure 1B), including sinuosity (a measure of the degree of interdigital webbing [27]), and foot shape derived from a set of nine landmarks and geometric morphometric methods [39-41]. The five species inhabiting Sardinia attain a larger body size and larger foot size than do species from continental Italy. Despite this, we found that adults of all species attain similar and relatively low values of sinuosity (${\bar{X}}_{range}$ = 2.38 to 2.73), implying that they display a high degree of interdigital webbing. Surprisingly however, when examined over the course of development, we found very different ontogenetic patterns among species. An analysis of covariance (ANCOVA) revealed that developmental trajectories differed among species (Table 1A), with most species displaying no change in foot webbing through ontogeny (Table 2). Thus, these species are best described as isometric, as their adult morphology was similar to that observed in juveniles. In stark contrast, three of the species from Sardinia (*H. (S.) flavus, H. (S.) sarrabusensis*, and *H. (S.) supramontis*) exhibited significant allometry, where the degree of interdigital webbing increased as animals grew larger (Table 2; Figure 2A, B). A multivariate analysis of covariance (MANCOVA) of foot shape also revealed species-specific allometric trajectories (Table 1B), consistent with the observations of foot webbing. As with the degree of foot webbing, most species displayed little change in foot shape with changes in size, again implying that adult morphology in these species was similar to that seen in juveniles. By contrast, the same three species from Sardinia exhibited considerable changes in foot shape across their ontogenetic trajectories (Figure 2C, D). For these species, adults have relatively wider feet with increased foot webbing, while juveniles have relatively narrower feet and less interdigital webbing (Figure 3).

**Table 1:** Results from statistical analyses of different components of foot morphology.

A) Foot Webbing	SS	MS	df	F	P
Species	4.5358	0.64797	7	30.9142	< 0.0001
Foot Length	0.5388	0.53881	1	25.7061	< 0.0001
Species × Foot Length	0.4830	0.06900	7	3.2919	0.0022


**B) Foot Shape**	**Pillai's Trace**	**Approx. F**	**df num**	**df den**	**P**

Species	1.84254	7.8341	98	2149	< 0.0001
Centroid Size	0.42077	15.6182	14	301	< 0.0001
Species × Centroid Size	0.46758	1.5696	98	2149	0.0004

A) The relative degree of foot webbing as a function of species and foot length. B) Foot shape from landmark-based geometric morphometrics as a function of species and foot size (centroid size).

**Table 2:** Species regressions of foot webbing versus foot length (bold-face indicates significant regression).

Species	β_1_	σ_β_	t	P
*H. (S.) ambrosii*	0.0001995	0.0066539	0.03	0.976 NS
***H. (S.) flavus***	**-0.03461**	**0.01234**	**-2.804**	**0.00708**
*H. (S.) genei*	-0.006958	0.010382	-0.67	0.51 NS
*H. (S.) imperialis*	-0.001060	0.006966	-0.152	0.88 NS
*H. (S.) italicus*	-0.008369	0.010279	-0.814	0.421 NS
***H. (S.) sarrabusensis***	**-0.05263**	**0.02109**	**-2.495**	**0.0199**
*H. (S.) strinatii*	0.001475	0.009891	0.149	0.882 NS
***H. (S.) supramontis***	**-0.03503**	**0.00878**	**-3.99**	**0.000208**

**Figure 4:**
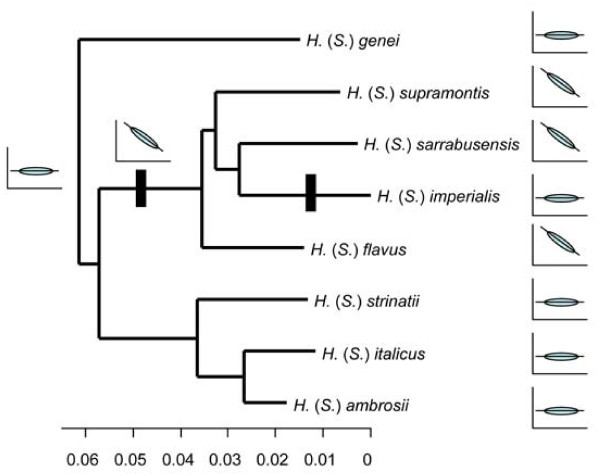
**Phylogenetic relationships for all species of European *Hydromantes***. Relationships based on a molecular phylogeny found from combined mitochondrial and nuclear DNA sequence data, with branch lengths proportional to nucleotide differences [28]. Observed patterns of foot allometry (Figure 2B) are denoted on the phylogeny, along with hypothesized evolutionary transitions found from maximum likelihood ancestral state reconstruction.

To better understand the evolution of these ontogenetic trajectories we used a recently published molecular phylogeny [28] and maximum likelihood ancestral character state reconstruction [42]. We found that isometry was the most likely ancestral condition for the European lineage, with a single evolutionary transition to an allometric growth pattern at the base of a clade containing four of the Sardinian species (Figure 4). However, one of these species, *H. (S.) imperialis*, displayed isometric foot growth, so an evolutionary reversal to isometry must also be proposed. Interestingly, the growth trajectory of one of the three North American species (*H. platycephalus*) has also been examined (M. Jaekel, pers. comm.), and this species exhibits allometric growth similar to that observed in the three Sardinian species. When *H. platycephalus *was included in the analysis, the patterns described above remained unchanged, but the ancestral condition for the entire genus was hypothesized to be allometric. This result implied that an evolutionary change to isometry occurred at the base of the lineage of European species (though if the remaining North American species also display variation in their growth patterns, the ancestral condition for *Hydromantes *would be harder to determine). Together, these analyses reveal that ontogenetic trajectories in *Hydromantes *are evolutionarily labile, with transitions between allometry and isometry occurring repeatedly within the lineage.

One striking feature of the observed interspecific ontogenetic trends was the fact that adults across species appeared more similar to one another than did juveniles (Figure 2). We performed a formal test of this observation, and found that the variation in the degree of interdigital webbing was significantly smaller in adults than it was in juveniles (∑*D*_*juv *_- ∑*D*_*adult *_= 3.3302; P_rand _= 0.0002). A similar finding was also obtained for variation in foot shape (∑*D*_*juv *_- ∑*D*_*adult *_= 0.8362; P_rand _= 0.0001). These results revealed that foot morphology among species was more similar in adults than it was among juveniles. Therefore, in contrast to tropical plethodontids whose differing growth trajectories resulted in divergent adult foot morphologies, the ontogenetic trajectories of European *Hydromantes *have converged on a common adult phenotype.

Finally, we examined allometric parameters of foot growth relative to a biomechanical model [26,27] to determine whether foot growth was adapted for climbing (see [27]). We found that foot growth parameters in the European plethodontid salamanders (α = 0.75, *b *= 0.14) were most similar to those of a tropical cave dwelling salamander, *Chiropterotriton magnipes *[27]. Interestingly, in a recent study of tropical plethodontids with extensive foot webbling, *C. magnipes *was the only species whose pattern of foot growth was adapted for climbing [27]. Thus, when compared to the growth parameters for this species, our findings suggest that patterns of foot growth in some European *Hydromantes *may also be adapted for climbing.

## Discussion

Understanding the evolution of morphological diversity requires a pluralistic approach, where both selective and structural mechanisms are investigated. In this study, we examined developmental trajectories of foot morphology in European plethodontid salamanders to determine what forces may have shaped patterns of morphological variation, and to test the hypothesis that adult foot morphology was an adaptation for climbing. Our results provide evidence that both selection and development influence patterns of foot morphology, implying a complex history of morphological evolution in the group.

First, our morphological analyses identified species-specific developmental trajectories of foot growth. Statistical models describing variation in foot morphology revealed a significant Species × Size interaction term (Table 1), indicating that patterns of foot growth were not consistent across all species of European *Hydromantes*. Upon further examination of these patterns (Table 2; Figure 2), we found that the species-specific patterns could be classified into two general categories. For most species of European *Hydromantes*, there was little change in the degree of foot webbing during growth, as both juveniles and adults displayed extensive foot webbing. Similar patterns were found in many tropical plethodontid species [27], which also displayed a high degree of interdigital webbing as adults. Thus, viewing our findings in light of previous results suggests that for these species of European *Hydromantes*, the extensive foot webbing observed in adults can be described as resulting from isometry during development (see discussion in [27]).

The remaining three species in our study displayed significant foot allometry, where the degree of interdigital webbing increased during growth (Table 2; Figure 2, Figure 3). This ontogenetic pattern may be expected under an adaptive scenario, given that a biomechanical model established that a greater degree of webbing and foot surface area is required for successful climbing as salamanders grow larger [26]. This hypothesis is further supported by the fact that for our species, the growth parameters of foot area relative to body size were most similar to those found in a tropical cave dwelling salamander (*C. magnipes*), whose foot growth was previously determined to be adapted for climbing [27]. These findings therefore provide some support for the hypothesis that foot morphology in the allometric European plethodontids is adaptive [31,34], and is, at least in part, a response to natural selection.

Surprisingly, despite the distinct ontogenetic trajectories in foot development observed in this lineage, patterns of adult foot morphology were strikingly similar among species. A formal test of this observation revealed that variation in adult foot morphology was significantly smaller than variation in juvenile foot morphology, implying that a common adult phenotype exists among species, despite large differences in their developmental trajectories. Such ontogenetic convergence was unexpected, as a study of tropical plethodontids found that different growth trajectories resulted in divergent adult foot morphologies among species [27]. Further, this ontogenetic convergence among species of European *Hydromantes *suggests that adult foot morphology may be more functionally constrained than that of juveniles, a hypothesis that was previously suggested for these species [34]. It also suggests that those species displaying allometric foot growth have overcome their initial unwebbed juvenile state via an evolutionary shift in their developmental trajectory, which results in an adult morphology with more extensive foot webbing. Indeed, functional experiments have shown that the ability of plethodontids to adhere to vertical surfaces is facilitated by increased foot surface area and increased webbing, particularly as animals grow larger [26]. Further, a biomechanical model demonstrated that clinging to vertical surfaces becomes more difficult as animals increase in size [26,27]. Based on these observations, it is reasonable to suggest that selection for climbing is more intense in adult European *Hydromantes *than it is in juveniles, and our observations of ontogenetic convergence are consistent with this interpretation.

When viewing these patterns in light of phylogeny, we found that ontogenetic growth trajectories were not static, and that multiple transitions between allometric and isometric growth had occurred during the evolutionary history of the group. This result implied that some degree of flexibility exists in the developmental program of these species, and that their ontogenetic trajectories are evolutionarily labile. However, all instances of allometry in this lineage followed a similar developmental trajectory, with an increase in foot webbing during growth. While the opposite pattern of decreased foot webbing during growth has been found in other plethodontids [27], this pattern was not observed in European *Hydromantes*. We hypothesize that this differences stems from the functional requirements for climbing. Our results suggest that there is a minimal degree of foot webbing (and thus foot area) required for European *Hydromantes *to climb successfully, and that this limit is more extreme for larger animals (see also [26,27]). The fact that different species arrive at a common adult phenotype from different juvenile morphologies lends further support to this hypothesis. Interestingly, the three species displaying allometric foot growth (*H. (S.) flavus, H. (S.) sarrabusensis*, and *H. (S.) supramontis*) are all geographically restricted species, and are found in more arid and rocky environments with a narrower range of available habitats as compared to the remaining species in the group (C. Corti, pers. comm.; A. Nistri, unpubl. data). Thus, selection on particular morphological traits may be accentuated in these species at all developmental stages due to their environmental condition. To date however, no quantitative, comparative, ecological studies have been performed of these species, which would provide critical evidence to test this hypothesis. Regardless, if this hypothesis is correct, it conforms with the previous suggestion that selection plays a role in shaping patterns of foot morphology in these salamanders [31,34]. The evolutionary changes seen in ontogenetic trajectories across the phylogeny for the group may therefore reflect the complex interplay between developmental processes on the one hand, and selective pressures that are more intense at a single developmental stage (adults).

Finally, we note that our findings differ from those of previous studies [27] in one important respect. In tropical *Bolitoglossa*, paedomorphic (isometric) and allometric trajectories diverge during growth to generate distinct adult phenotypes (webbed and unwebbed adults respectfully: [27]), while in European *Hydromantes*, isometric and allometric growth trajectories converge on a common adult morphology with extensive foot webbing. This distinct pattern of interspecific convergence provides strong evidence of selection on adult foot morphology in European *Hydromantes*, and raises a number of interesting evolutionary questions for future work. For instance, because both juveniles and adults of all European *Hydromantes *climb successfully, and are found in similar habitats, it is of interest to determine whether the juveniles of isometric species are 'over-engineered' for climbing, or whether there is a cost to having more extensive foot webbing as compared to juveniles of other European *Hydromantes *species. In addition, the fact that ontogenetic patterns of foot growth are so evolutionarily labile in *Hydromantes *begs the question of what may be responsible for such lability, and how the genetic underpinnings of these traits affect the ontogenetic patterns. When viewed in light of the biogeography of the species, our patterns imply that perhaps the evolutionary changes in ontogenetic trajectories are associated with specific distributional changes of the species, such as founder events or the colonization of new habitats. Finally, the fact that both isometric and allometric growth patterns converge on a common (selectively advantageous) foot morphology implies that both developmental trajectories may be selectively beneficial, and suggests that in this instance, multiple developmental solutions exist to the same evolutionary challenge.

## Conclusions

This study characterized ontogenetic trajectories of foot morphology in all eight species of European plethodontid cave salamanders to test the hypothesis that adult foot morphology was an adaptation for climbing. We showed that five of the eight species displayed little change in foot morphology during growth (isometry), while the remaining species showed an increase in foot webbing as animals grew larger. Despite these different developmental trajectories however, we also showed that adult foot morphology converged on a common phenotype across species, suggesting that functional demands for vertical climbing are more intense in adults than in juveniles. We further showed that growth patterns were consistent with selection for improved climbing. The findings presented here demonstrate that both selection and developmental processes have influenced phenotypic evolution in this group.

## Methods

A total of 330 salamander specimens from the collections of the Museo di Storia Naturale (Sezione di Zoologia), Universitá di Firenze (MZUF) were used in this study (mean = 41; range = 24 - 54). To capture ontogenetic information, we included a wide size range of juveniles and adults for each species (these species are direct-developers with no larval stage). Only specimens from a single geographic locality per species were utilized to minimize among-locality variability. We characterized foot morphology using several different quantitative measures. All measurements were taken from the right foot of each specimen (with the exception of a few specimens whose right foot was poorly preserved). First, we used a sinuosity measure, which quantified the degree of interdigital webbing for each individual [27]. Here the perimeter of the distal portion of the foot was measured as the outline from the tip of digit one to digit five, and sinuosity was measured as the ratio of this perimeter relative to the width of the foot (p/d: Figure 1B). Sinuosity decreases as the degree of interdigital webbing increases. Second, we quantified foot shape using geometric morphometric methods [39-41]. These methods quantify the shape of anatomical objects from the coordinates of repeatable locations, after non-shape variation has been mathematically held constant (e.g., [9,43,44]). For this approach, the locations of nine anatomical landmarks were recorded from the foot of each specimen (points 1-9: Figure1B). Landmark configurations for each specimen were then optimally aligned using a generalized Procrustes superimposition [45], and from the aligned specimens, Procrustes tangent coordinates were used as a set of shape variables for all multivariate analyses. The centroid size for each foot was also retained for further analysis. Finally, we measured total foot area for each specimen (A: Figure 1B), as well as the body weight for those specimens that were fully intact (some specimens could not be weighed because some limbs had been previously removed for other investigations, or because they were overly dry due to preservation).

We performed a number of statistical analyses to examine the ontogeny of foot morphology. First we used an analysis of covariance (ANCOVA) to compare allometric trends in foot webbing across species, using species, foot width, and their interaction as model effects. We also conducted linear regressions of the degree of foot webbing versus foot width for each species separately. Next, we performed a multivariate analysis of covariance (MANCOVA) to compare allometric trends in foot shape, using species, centroid size, and their interaction as model effects. Multivariate patterns of ontogenetic change were visualized using scores from a multivariate regression of foot shape versus log(Centroid Size) [46], and thin-plate spline deformation grids [47] were used to facilitate biological interpretation of allometric changes in foot shape. Plots of predicted values from species-specific multivariate regressions were also generated to facilitate species-level comparisons.

To determine whether ontogenetic trajectories in foot morphology converged on similar adult morphologies, we performed a permutation procedure that compared variation in juveniles to variation in adults among species. With this approach, predicted morphologies along each species' ontogenetic trajectory were estimated for all specimens, and the predicted morphologies for the smallest and largest observed specimen in each species were obtained. Next, the Euclidean distances between all pairs of small specimens and between all pairs of large specimens were calculated. These distances were then summed, and the test statistic (∑*D*_*juv *_- ∑*D*_*adult*_) was calculated. A positive test statistic implied that adults were more similar to one another than were juveniles, while a negative test statistic implied that juveniles were more similar to one another than were adults. Finally, the observed test statistic was compared to a distribution of possible values obtained through permutation. Here, the predicted morphologies for all individuals were randomized with respect to their size, new predicted morphologies at small and large values were obtained, and a new test value was calculated. The proportion of randomly generated test values (of 9,999) larger or equal to the observed was taken as the significance level (for a related approach comparing morphological trajectories, see [48-50]).

Evolutionary transitions in ontogenetic growth were estimated using maximum likelihood ancestral state reconstruction [42]. A recent molecular phylogeny [28], containing all eleven species of *Hydromantes *and several additional taxa was used in these analyses. First we pruned the phylogeny so that a single population per European *Hydromantes *species was retained (populations corresponded geographically to those locations from which we obtained our morphological data). We then coded each ontogenetic trajectory as 'allometric' or 'isometric', based on whether or not the species-specific regression of sinuosity versus foot length was significant. We then used discrete-based maximum likelihood [42] to estimate ancestral states for each internal node of the tree.

Finally, we quantified allometric patterns of foot growth to determine whether they conformed to adaptive expectations based on a prior biomechanical model [26,27]. Here, total foot area (A) was modeled as a function of body weight (W) using the allometric equation: *A *= *bW*^α^. From this the allometric growth parameters (α and b) were obtained, where b represents the intercept and α is the relative growth parameter. These were then compared to values previously identified for other plethodontid salamanders [27]. Prior work had shown that one tropical species, the cave-dwelling *C. magnipes*, exhibited foot morphology adapted for climbing and clinging to vertical surfaces, while other tropical, arboreal species (genus *Bolitoglossa*) did not display adaptive patterns. We therefore compared parameters for European *Hydromantes *to these species, to determine which species more closely matched the observed foot ontogeny in this group.

All statistical analyses were performed in R 2.10.1 [51].

## Authors' contributions

DCA designed the study, contributed to data collection, performed statistical analyses, and wrote the manuscript. AN collected the data, participated in statistical analyses, and assisted in writing the manuscript. All authors read and approved the final manuscript.
